# Corneal abrasions in space: current therapeutics and future directions

**DOI:** 10.1038/s41433-023-02911-3

**Published:** 2024-01-04

**Authors:** Ethan Waisberg, Joshua Ong, Andrew G. Lee

**Affiliations:** 1 Department of Ophthalmology, University of Cambridge, Cambridge, UK; 2 Michigan Medicine, University of Michigan, Ann Arbor, MI USA; 3 Center for Space Medicine, Baylor College of Medicine, Houston, TX USA; 4 Department of Ophthalmology, Blanton Eye Institute, Houston Methodist Hospital, Houston, TX USA; 5 The Houston Methodist Research Institute, Houston Methodist Hospital, Houston, TX USA; 6 Departments of Ophthalmology, Neurology, and Neurosurgery, Weill Cornell Medicine, New York, NY USA; 7 Department of Ophthalmology, University of Texas Medical Branch, Galveston, TX USA; 8 University of Texas MD Anderson Cancer Center, Houston, TX USA; 9 Texas A&M College of Medicine, Bryan, TX USA; 10 Department of Ophthalmology, The University of Iowa Hospitals and Clinics, Iowa City, IA USA

**Keywords:** Health care, Risk factors

While spaceflight associated neuro-ocular syndrome (SANS) is the most commonly discussed concern affecting astronaut vision during long duration spaceflight, ocular trauma represents a significant, yet underappreciated ophthalmic risk during space missions [1, 2]. The National Aeronautics and Space Administration Human Research Program (NASA HRP) previously identified corneal abrasion as having a “high likelihood of occurrence” and “severe consequence” if no treatment was available [3]. With the commercialization of spaceflight, and expected exponential rise in space travel in the coming years, the frequency of ocular injuries will likely increase [4, 5]. Meer et al. [6] reviewed ocular trauma during NASA space missions and described 70 prior corneal abrasions. The future construction needs for lunar and orbital habitats will also likely increase the risk and exposure for corneal abrasions, ocular trauma, and foreign bodies.

The cornea is a delicate structure that plays an essential role in focusing light that enters the eye to maintain clear vision but is also exposed to the risks of trauma from the external environment. During spaceflight, unique ocular hazards may be encountered by astronauts which could potentially compromise corneal integrity and threaten mission critical activities. In this paper we examine the unique factors in spaceflight that predispose individuals to corneal and ocular surface disease and trauma; discuss potential countermeasures to mitigate these risks; and possible treatment options.

## Microgravity

The risk of an ocular injury from a foreign body in space and microgravity may be higher than on Earth because unintended airborne particulate matter remains suspended and may travel along existing air currents in the space craft [7]. Microgravity also interferes with tear distribution across the cornea, which can potentially lead to increased dryness and thus further increase susceptibility to abrasions. Additionally, research in mice has shown that the cephalad fluid shifts that occur in microgravity predispose to the development of corneal oedema during long-duration spaceflight (LDSF) [8].

## Celestial debris

With future planned missions to the Moon and Mars, understanding the ophthalmic risks of exposure to dust from celestial bodies is essential. The Moon and Mars are both covered by a loose rock (known as regolith), and abrasive dust [6]. Lunar and Martian windstorms and other environmental factors unique to space may also further increase the risk of exposure to corneal injury. During the Apollo missions, astronauts reported both eye and skin irritation due to lunar dust [6]. In addition to mechanical irritation, prolonged exposure to low levels of lunar dust has been shown to elicit molecular responses in cornea tissue, potentially affecting mitochondrial dysfunction, epithelial healing, oxidative stress response, eye development and cellular proliferation [9].

## Treatment

On Earth, foreign bodies in the conjunctiva or cornea can be removed by irrigation with saline or for larger corneal foreign bodies with the use of sterile corneal spuds. Particles of space dust may be particularly challenging to visualize without the use of slit lamp biomicroscopy (not currently available on the space station). Moreover, more serious ocular infections including conjunctivitis and microbial keratitis can be mission threatening on the International Space Station (ISS). On ISS, current protocols for treatment include first line therapy with topical antibiotic (e.g., polytrim (polymyxin B/ trimethoprim) solution) [6] and if no improvement is seen, then therapy is advanced to topical ciprofloxacin (1 drop in the affected eye, every 6 h) [6]. Early recognition and treatment of ocular infections is vital during spaceflight, particularly due to alterations in virulence and enhanced microbial growth that is generally seen in space [10]. Immunosuppression has also been reported in astronauts due to a multitude of stressors encountered in spaceflight, which further emphasizes the danger posed by eye infections in space [11].

Further research will be essential to mitigate the risk of corneal abrasions in spaceflight. Artificial tears can potentially be used daily by astronauts to ensure that the ocular surface remains adequately lubricated. Omega 3 fatty acid supplementation has also previously been investigated for dry eye disease, and may be useful for astronauts to minimize ocular surface irritation [12]. Additionally, further vision testing is required to ensure that any astronaut vision decrements are detected early, to minimize ophthalmic risks [13–15].

## References

[1] Ong J, Tavakkoli A, Zaman N, Kamran SA, Waisberg E, Gautam N (2022). Terrestrial health applications of visual assessment technology and machine learning in spaceflight associated neuro-ocular syndrome. npj Microgravity.

[2] Ong J, Waisberg E, Masalkhi M, Kamran SA, Lowry K, Sarker P (2023). Artificial intelligence frameworks to detect and investigate the pathophysiology of spaceflight associated neuro-ocular syndrome (SANS). Brain Sci.

[3] Eye Abrasion (Foreign Body). NASA Human Research Roadmap. Accessed November, 2023. https://humanresearchroadmap.nasa.gov/Evidence/medicalConditions/Eye_Abrasion_(Foreign_Body).pdf.

[4] Waisberg E, Ong J, Masalkhi M, Lee AG, Berdahl J. Anatomical considerations for reducing ocular emergencies during spaceflight. Ir J Med Sci. Published online May, 2023. 10.1007/s11845-023-03407-5. 10.1007/s11845-023-03407-5 PMC10808690 37243845

[5] Waisberg E, Ong J, Masalkhi M, Zaman N, Kamran SA, Sarker P, et al. The case for expanding visual assessments during spaceflight. Prehosp Disaster Med. Published online June, 2023:1–4. 10.1017/S1049023X23005964. 10.1017/S1049023X23005964 PMC10445111 37365808

[6] Meer E, Grob S, Antonsen EL, Sawyer A (2023). Ocular conditions and injuries, detection and management in spaceflight. npj Microgravity.

[7] Barratt MR, Baker E, Pool SL, eds. *Principles of Clinical Medicine for Space Flight*. 2nd ed. Springer Springer [Distributor]; 2020.

[8] Zanello SB, Theriot CA, Ponce CMP, Chevez-Barrios P (2013). Spaceflight effects and molecular responses in the mouse eye: preliminary observations after shuttle mission STS-133. Gravitational Space Res.

[9] Theriot C, Glass A, Lam C, James J, Zanello S. Chronic lunar dust exposure on rat cornea: evaluation by gene expression profiling. Accessed November, 2023. https://ntrs.nasa.gov/search.jsp?R=20140003859.

[10] Horneck G, Klaus DM, Mancinelli RL (2010). Space microbiology. Microbiol Mol Biol Rev.

[11] Klaus DM, Howard HN (2006). Antibiotic efficacy and microbial virulence during space flight. Trends Biotechnol.

[12] Kawakita T, Kawabata F, Tsuji T, Kawashima M, Shimmura S, Tsubota K (2013). Effects of dietary supplementation with fish oil on dry eye syndrome subjects: randomized controlled trial. Biomed Res.

[13] Sarker P, Ong J, Zaman N, Kamran SA, Waisberg E, Paladugu P (2023). Extended reality quantification of pupil reactivity as a non-invasive assessment for the pathogenesis of spaceflight associated neuro-ocular syndrome: A technology validation study for astronaut health. Life Sci Space Res.

[14] Waisberg E, Ong J, Paladugu P, Kamran SA, Zaman N, Lee AG (2023). Dynamic visual acuity as a biometric for astronaut performance and safety. Life Sci Space Res.

[15] Waisberg E, Ong J, Zaman N, Paladugu P, Kamran SA, Tavakkoli A (2023). The spaceflight contrast sensitivity hypothesis and its role to investigate the pathophysiology of spaceflight-associated neuro-ocular syndrome. Front Ophthalmol.

